# *Besnoitia tarandi* in Canadian woodland caribou – Isolation, characterization and suitability for serological tests

**DOI:** 10.1016/j.ijppaw.2018.11.005

**Published:** 2018-11-27

**Authors:** Gereon Schares, Charles Jutras, Andrea Bärwald, Walter Basso, Aline Maksimov, Susann Schares, Mareen Tuschy, Franz J. Conraths, Vincent Brodeur

**Affiliations:** a Friedrich-Loeffler-Institut, Federal Research Institute for Animal Health, Institute of Epidemiology, Südufer 10, 17493, Greifswald - Insel Riems, Germany; b Direction de la gestion de la faune du Nord-du-Québec, Ministère des Forêts, de la Faune et des Parcs du Québec, 951 boul. Hamel, Chibougamau, Québec, G8P 2Z3, Canada; c Institute of Parasitology, University of Bern, Länggassstrasse 122, 3012, Bern, Switzerland

**Keywords:** *Besnoitia tarandi*, *In vitro* isolation, Multilocus microsatellite typing, Serological assay

## Abstract

In the present study, we report the first *in vitro* isolation of *Besnoitia tarandi* from North America and the second of *B. tarandi* at all. The parasite was isolated directly from the skin of a Canadian woodland caribou from the migratory ecotype. The animal belonged to the Leaf River Herd, in Northern Quebec, Canada. The isolate was designated Bt-CA-Quebec1.

Sequencing of the 3’-end of the 18S rRNA gene, the complete sequence of the ITS1 and the 5’-end of the 5.8S rRNA gene of Bt-CA-Quebec1 revealed only minor differences to rDNA gene fragments of *B. besnoiti*. In contrast, the patterns for the microsatellite loci Bt-20 and Bt-21 varied substantially from those reported for *B. besnoiti* and *B. bennetti.* Surprisingly, the typing results in the loci Bt-6 and Bt-7 differed between Bt-CA-Quebec1 and results obtained for skin samples from caribou of the Canadian regions of Nunavut and the Northwest Territories reported by other investigators. This indicates that differences might exist among *B. tarandi* in caribou from different regions in Canada.

Mice (γ-interferon knockout) intraperitoneally inoculated with 1.2 × 10^6^ or 1.5 × 10^6^ bradyzoites mechanically released from skin tissue cysts fell ill 8, 9 or 18 days post inoculation. GKO mice inoculated with 3.0 × 10^4^ tachyzoites isolated from the peritoneal cavity of a bradyzoites-inoculated mouse became ill earlier, i.e. 5 days post inoculation. Lung was the predilection site in all mice.

Bt-CA-Quebec1 tachyzoites rapidly grew in MARC-145 cells and were used for antigen production. Comparative Western blot analyses revealed only a few differences between *B. tarandi* Bt-CA-Quebec1 and *B. besnoiti* Evora antigen when probed with sera collected from chronically infected caribou.

Due to its fast growth *in vitro*, the Bt-CA-Quebec1 isolate may represent an interesting antigen source to establish *B. tarandi*-specific serological tools and to study the biology of this parasite species further.

## Highlights

• *Besnoitia tarandi* was *in vitro-*isolated directly from the skin of a Canadian woodland caribou.
• Microsatellite typing revealed differences to existing Bt-6, Bt-7 and Bt-21 patterns of *B. tarandi*.
• The isolate Bt-CA-Quebec1 caused disease in γ-interferon knockout mice.
• Bt-CA-Quebec1 rapidly grows in MARC-145 cells.
• Antigens of Bt-CA-Quebec1 are similar but not identical to those of *Besnoitia besnoiti*.

## Graphical abstract

**Figure:**
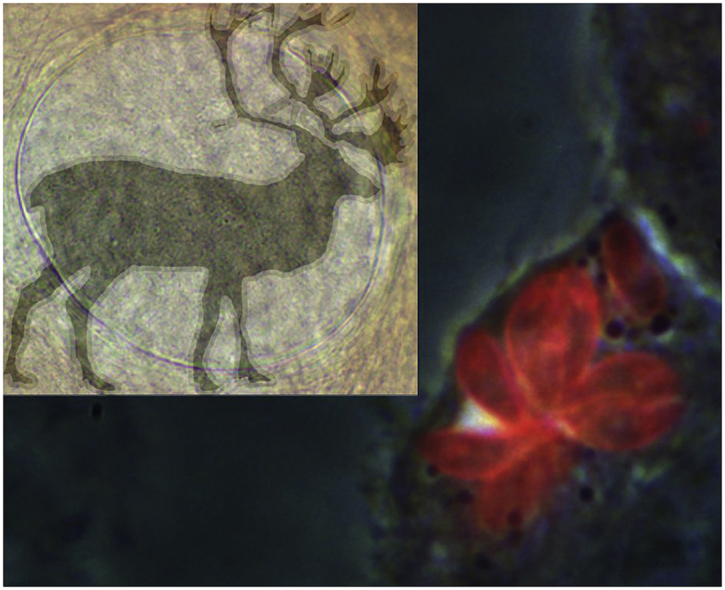

## 1 Introduction

Caribou (*Rangifer tarandus caribou* and other caribou subspecies) and reindeer (*Rangifer tarandus tarandus*) are known as intermediate hosts of the protozoan parasite *Besnoitia tarandi* (Dubey et al., 2004; Madubata et al., 2012). A first description of the disease in Alaskan reindeer and caribou is from 1922 (Hadwen, 1922). Meanwhile, disease descriptions are available for many regions of the arctic and subarctic zones including North American, European and Asian regions and based on a microscopic and gross visual assessment Canadian mule deer (*Odocoileus hemionus*) and muskoxen (*Ovibos moschatus*) also seem to suffer from *Besnoitia* spp. infections as summarized by Olias et al. (2011). The disease is characterised by skin changes predominantly affecting the distal extremities, eyelids, periorbital skin, or lips (alopecia, hyperpigmentation, thickening, fissuring, ulceration and generalized exudative dermatitis). Characteristic tissue cysts (about 0.5–1 mm in size) with a thick secondary cyst wall (Dubey et al., 2004; Hilali et al., 1990) are often observed in large numbers in the dermis (Ducrocq et al., 2012). In addition to dermal tissue, turbinate mucosae and scleral conjunctivae as well as subcutaneous tissues, peritendinous fasciae and the periosteum represent frequent cyst locations (Dubey et al., 2004; Ducrocq et al., 2012; Glover et al., 1990). In severe cases, the animals may become cachectic (Wobeser, 1976) and in analogy to bovine besnoitiosis, it seems possible that males may become sterile (Kumi-Diaka et al., 1981). However, heavily affected animals represent a minority and recent findings suggest that gross examinations often miss existing infections because they are subclinical and are not detected during visual inspection (Ducrocq et al., 2012). To estimate the prevalence more accurately, serological surveys with sensitive and specific diagnostic tests need to be performed.

The size of several caribou herds decreased dramatically during past years (Gunn et al., 2011), which is still undergoing for the Leaf River and George River Herds in Northern Quebec and Labrador (Brodeur et al., 2018; Taillon et al., 2016). Parasites are known to influence population dynamics of caribou (Albon et al., 2002; Hughes et al., 2009). There is evidence that heavy *Besnoitia* infections in caribou can be associated with a lower winter survival rate (Ducrocq et al., 2013). The prevalence of infected caribou in the Leaf River Herd (based on the histological analysis of metatarsal skin samples) increased from approximately 30% to over 80% between 2007 and 2011 (Taillon et al., 2016) and this trend is expected to have been similar but lower in the George River Herd. Between 2006 and 2009, the rapid increase of the *B. tarandi* prevalence in caribou coincided with a reduction of 58% of the proportion of large males that was synchronous in each population (based on data made available to VB by the Government of Quebec and the Government of Newfoundland and Labrador). Intriguingly, *B. tarandi* had not been identified in Quebec and Labrador prior to 2006, and was therefore not a concern in the monitoring of local caribou populations. The impact of *B. tarandi* infections on the dynamics of the caribou populations may be significant, but it is currently not known, to which extent and by which mechanisms (Ducrocq et al., 2013).

Although a two-host life-cycle is assumed for *B. tarandi* suggesting at least one carnivorous animal as a definitive host, e.g. a large felid like cougar or lynx (Dubey et al., 2004), neither the definitive host nor the parasitic stages shed with the faeces by this host, the oocysts, are known. Feeding of fresh caribou tissues containing large numbers of *Besnoitia* sp. cysts to juvenile, zoo raised raccoons, house-raised cats, laboratory dogs and one juvenile zoo-raised arctic fox failed to induce oocysts shedding in these animals (Ayroud et al., 1995; Dubey et al., 2004; Glover et al., 1990). This situation is similar to that in other closely related apicomplexan species such as *Besnoitia besnoiti* (cattle are intermediate hosts), *Besnoitia caprae* (goats are intermediate hosts) and *Besnoitia bennetti* (donkeys and horses are intermediate hosts), i.e. *Besnoitia* sp., for which the definitive host is also unknown (Basso et al., 2011). Similar to the situation in cattle, it has been assumed that the predominant route of infection is horizontal, i.e. mechanical by the mouth parts of biting insects, e.g. by tabanids or *Stomoxys* sp. from an infected to a non-infected caribou (Ducrocq et al., 2013). The importance of biting insects for the transmission between animals is likely because higher densities of *B. tarandi* cysts were found in caribou in late summer or autumn (i.e. shortly after the insect season) compared to the following early summer season (Ducrocq et al., 2013). Whether natural mating represents another route of horizontal transmission of *Besnoitia* spp. is under discussion, but specific evidence is lacking in caribou, similar to the situation in bovine besnoitiosis (Gollnick et al., 2015).

The aim of the present study was to establish a first *in vitro*-isolate of *B. tarandi* from migratory caribou in North America and to characterise this isolate relative to existing *B. tarandi* of reindeer and to closely related *Besnoitia* sp. of cattle and donkeys, i.e. *B. besnoiti* and *B. bennetti*. In addition, we aimed at exploring the *in vitro*-growth characteristics of the new isolate to assess its potential suitability as an antigen source for serological investigations.

## 2 Material and methods

### 2.1 Source and processing of skin samples

On December 6th, 2011, skin portions of three different hunted woodland caribou of the migratory ecotype (Courtois et al., 2003) showing macroscopical signs of besnoitiosis (i.e. skin nodules) were submitted to the Friedrich-Loeffler-Institut, Institute of Epidemiology, Wusterhausen, Germany. All samples had been collected from female adult animals, designated H-1145, H-1146 and H-1148, which had belonged to the Leaf River Herd, Quebec, Canada. They had been hunted at 53°41′21″ N 73°20′03″ W (NAD83)). In case of H-1145 and H-1146, the skin sample was from the rostrum. In case of H-1148, the sample had been obtained from the skin of the hind foot, in front of the metatarsus.

Samples arrived late in the evening of December 9th, 2011. They were stored in a refrigerator until December 12th, 2011. To remove accidental surface contaminations and haired parts from skin, the external parts of the samples were trimmed away. The cores (about 25–50 mg) were squashed using a mortar and pestle in 1 ml Dulbecco's Modified Eagle Medium (DMEM) supplemented with 2% foetal calf serum (FCS), 1% antibiotic solution (10,000 IU penicillin and 10,000 μg streptomycin/ml solution) and 1% amphotericin B (250 μg/ml).

**Fig. 1:**
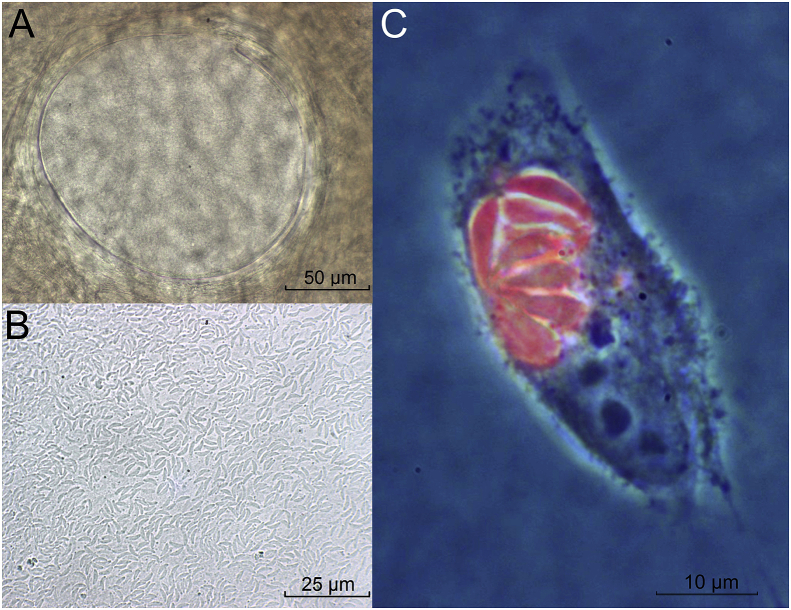
*Besnoitia* from Canadian woodland caribou. (A) *Besnoitia tarandi* tissue cyst in caribou skin (animal H-1145), (B) mechanically released bradyzoites used for mouse or cell culture inoculation and (C) a parasitophorous vacuole in a MARC-145 cell stained by immunofluorescence using a rabbit anti-*Besnoitia besnoiti* antibody.

The suspensions and the squashed skin samples were examined by light microscopy (100, 200 and 400× magnification) to confirm the presence of bradyzoites and to screen for intact or broken tissues cysts (Fig. 1A). Since microscopic inspection revealed that many bradyzoites had remained trapped in tissue cysts (Fig. 1A), small parts of the squashed samples were subjected to standard pepsin digestion as described (Dubey, 1998; Schares et al., 2017a). The number of bradyzoites released mechanically or after pepsin digestion were determined in the suspensions obtained using a Neubauer chamber. Bradyzoites were inoculated into cell cultures or intraperitoneally (ip) into mice in varying doses (Table 1).

**Table 1:** Inoculation of γ-interferon knockout mice with *Besnoitia tarandi* bradyzoites obtained from the skin of a Canadian caribou.

Mouse	Caribou	Treatment of tissue	Inoculation dose	Route of inoculation	End of experiment (dpi)	PCR^d^ result
K280-1	H-1145	Mechanical	1.2 E+06^a^	ip^b^	9^c^	Positive
K280-2	H-1145	Mechanical	1.2 E+06	ip	8^c^	Positive
K281-1	H-1146	Mechanical	1.5 E+06	ip	18^c^	Positive
K281-2	H-1146	Mechanical	1.5 E+06	ip	42	ND^e^
K282-1	H-1145	Mechanical + Pepsin	1.5 E+06	ip	42	ND
K282-2	H-1145	Mechanical + Pepsin	1.5 E+06	ip	42	ND
K283-1	H-1145	Peritoneal parasites of K280-2	3.0 E+04	ip	5^c^	Positive
K283-2	H-1145	Peritoneal parasites of K280-2	3.0 E+04	ip	5^c^	Positive

a 1 × 10^4^ bradyzoites of this preparation were also inoculated on MARC145 cell culture.

b Intraperitoneal.

c Experiment was terminated due to signs of disease (ruffled coat, lethargy).

d For details see Table 2.

e ND = Not determined.

### 2.2 Caribou sera

To assess the potential suitability of isolated parasites for diagnostic purposes, sera were collected from Canadian wild living female caribou in March 2012 and October 2013 (Table 2). Visual inspection of sclera and skin had revealed signs of chronic besnoitiosis in four of these migratory caribou, captured in 2013. In addition, five sera had been taken in 2012 from woodland caribou of the sedentary ecotype that did not show signs of chronic besnoitiosis and that were from a population expected to be free of the parasite. All sera were shipped to the Institute of Epidemiology, Friedrich-Loeffler-Institut, Greifswald-Insel Riems, Germany. Sera were further characterised using *B. besnoiti* as an antigen and as previously described, by APure-BbELISA (Schares et al., 2013) and by IFAT (Schares et al., 2010). All sera were tested by immunoblot for antibodies against *Neospora caninum* and *Toxoplasma gondii* with a negative outcome (Supplementary data, Fig. S1).

**Table 2:** Origin and serological characteristics of sera collected from female Canadian caribou with and without signs of chronic besnoitiosis.

Animal designation	Capture date	Eco-type	Serum designation	Clinical signs of chronic besnoitiosis	ELISA index in APure-BbELISA (cut-off 1.7)	IFAT titre using *B. besnoiti* Bb-EvoraCl2 as an antigen
BCN-01	March 2012	Sedentary	P15/4854	No	0.281	<1:50
BCN-03	March 2012	Sedentary	P15/4855	No	0.232	<1:50
BCN-07	March 2012	Sedentary	P15/4856	No	0.237	<1:50
BCN-09	March 2012	Sedentary	P15/4857	No	0.204	<1:50
BCN-11	March 2012	Sedentary	P15/4858	No	0.367	<1:50
F217	October 2013	Migratory	P15/4859	Yes	4.332	1:3200
F216	October 2013	Migratory	P15/4860	Yes	4.008	1:800
F211	October 2013	Migratory	P15/4862	Yes	4.140	1:800
F208	October 2013	Migratory	P15/4863	Yes	3.865	1:400

### 2.3 Mouse experiments

For bioassay, γ-interferon knockout mice (C.129S7 (B6)-Ifngtm1Ts/J, The Jackson Laboratory, Bar Harbor, Maine, USA) were inoculated ip in varying doses (Table 1). The animals were clinically observed for up to 42 days, bled on the day of necropsy or 42 days post inoculation and examined by immunoblotting (Schares et al., 2010). If the animals showed signs of disease (ruffled coat, lethargy), they were sacrificed immediately. All animals were necropsied except those that had neither seroconverted nor developed disease until the end of the experimental period.

### 2.4 Cell culture and isolation of tachyzoites

MARC-145 cells (rhesus monkey (*Macaca mulatta*), foetal kidney cells, permanent) were maintained in DMEM, 2% FCS at 37 °C/5% CO_2_. Depending on the growth rate, MARC-145 cells were split once or twice every two weeks. Confluent MARC-145 cells were infected with non-standardized doses of *B. besnoiti* (Bb1Evora03 (Cortes et al., 2006) and an as yet unpublished clone of Bb1Evora03, designated Bb-EvoraCl2) or *B. tarandi*. One day before harvesting the parasites for antigen production, the foetal calf serum (FCS)-supplemented medium was removed and infected cells were further cultivated under FCS-free conditions to avoid that *N. caninum* specific antibodies frequently present in FCS may cause false positive reactions in tests based on antigen not generated FCS-free (Dubey et al., 2017). After scraping the cells, parasites were purified by filtration using 5 μm hydrophilic syringe filters (Sartorius Lab Instruments, Göttingen, Germany). Parasites were washed 5 times by centrifugation at 700×*g* (4 °C, 8 min) and re-suspended in ice-cold phosphate buffer saline (PBS). Tachyzoites were then frozen as a pellet at −80 °C until used for Western blotting, IFAT or DNA extraction.

*In vitro* multiplication of *Besnoitia* tachyzoites in MARC-145 was preliminary assessed in 24 well plates (seeded with 1*10^5^ MARC-145 cells on the previous day) similar to previously described experiments (Frey et al., 2016). Briefly, seeded cells were inoculated on day 0 with 1 × 10^6^ tachyzoites of *B. besnoiti* Bb-EvoraCl2 or *B. tarandi* Bt-CA-Quebec1 and after 4 h of incubation (invasion time), the supernatant was removed and each well flushed 3 times with fresh cell culture medium. After 24, 48 or 72 h, the experiment was terminated, and the cells released mechanically by a rubber policeman into the medium supernatant. After transfer into a clean reaction tube, the suspension of cells in medium was centrifuged at 1800×*g*, the supernatant discarded, and DNA extracted from the pellet. Each sample (i.e. 3 samples per parasite and time period) was quantified for *B. besnoiti* DNA by using the BbRT2 real-time PCR (Schares et al., 2011). Ct values were assessed by linear regression using the respective function in the Microsoft EXCEL spreadsheet. At the time of this experiment, *B. tarandi* Bt-CA-Quebec1 was exactly 365 days in culture while *B. besnoiti* Bb-EvoraCl2 had been in culture for more than 5 years.

### 2.5 DNA isolation and real-time PCR to assess *Besnoitia* infection

DNA was extracted from bovine skin samples, GKO mouse tissues (brain, heart, lung, liver, spleen, kidney, skeletal muscle, skin), *in vitro*-grown tachyzoites and cell cultures with a commercial kit (NucleoSpin^®^ Tissue, Macherey-Nagel, Düren, Germany) according to the manufacturer's instructions. *B. tarandi* (Bt-CA-Quebec1) parasites were collected from MARC 145 cell cultures about one month after cell culture had been inoculated with bradyzoites.

The real-time PCR on *Besnoitia* was performed using the BbRT2 protocol with the primers Bb3 and Bb6 and the probe Bb3–6 (5’-FAM, 3’-BHQ1) as described by Schares et al. (2011). This PCR covers all *Besnoitia* species of ungulates as shown previously (Schares et al., 2011).

### 2.6 Immunofluorescence analysis

A serum collected 11 weeks post infection from a rabbit inoculated subcutaneously (sc) with *B. besnoiti* tachyzoites (Basso et al., 2011) was used to show *B. tarandi* tachyzoites in infected cell cultures as published (Schares et al., 1999). The pre-inoculation serum of this rabbit was used as a negative control. To analyse intracellular *B. tarandi* isolated from caribou skin, MARC-145 cells were grown on 12 mm coverslips and used after 1 day post infection (dpi). Cells were infected with about 1.0 × 10^5^ tachyzoites per coverslip. After 1 day, the coverslips were washed once in PBS and the cells fixed for 10 min by 2% paraformaldehyde and 0.05% glutardialdehyde in PBS. After washed in PBS, the cells were incubated with 50 mM ammonium chloride in PBS for 5 min, washed in PBS again and soaked two times in freshly prepared sodium borohydride solution (1 mg ml^−1^) for 5 min followed by permeabilization with 0.2% Triton X-100 in PBS for 20 min. Prior to the incubation with the polyclonal rabbit antibody (dilution 1:100), the cell cultures were blocked with 0.1% Triton X-100 and 2% gelatine in PBS for 15 min. After removing the blocking solution, the serum dilution was added. Blocking solution was used as a negative control. Subsequently, the wells were gently washed with PBS. Bound antibodies were detected after incubation with Texas Red conjugated anti-rabbit IgG [H + L] (Dianova) diluted 1:500. After further washings with PBS, the coverslips were mounted using Mowiol (Calbiochem) and examined with a Vanox AHBT3 fluorescence microscope (Olympus).

### 2.7 Sequencing of internal transcribed spacer (ITS) 1 and 5.8S and 18S rDNA

In addition to the primer pairs Tim2/Tim11 and Tim3/Tim11 used in other studies to characterise tissue cyst-forming coccidia (Schares et al., 2005, 2008b), previously described *Besnoitia* spp.-specific primer pairs were employed (Schares et al., 2009). The primer pairs BbGS1F/BbGS1R, BbGS2F/BbGS2R, BbGS3F/BbGS3R, BbGS4F/BbGS4R, BbGS5F/BbGS5R, BbGS6F/BbGS6R, BbGS6F/BbGS5R, BbGS2F/BbGS4R, BbGS3/TIM11 were used to generate multiple overlapping amplicons of parts of the 18 S rDNA, the ITS1 and parts of 5.8 S rDNA. PCR primers were used at a final concentration of 0.5 μM and dNTPs at a final concentration of 250 μM each (Amersham Biosciences, Piscataway, USA). DyNAzyme II DNA polymerase, (Finnzymes, Espoo, Finland) was added at 1 U/25 μl with the provided buffer. The reaction mix was supplemented with bovine serum albumin (lyophilized powder, suitable for molecular biology, non-acetylated, Sigma-Aldrich, Taufkirchen, Germany) at a concentration of 20 μg/ml 1 μl of genomic DNA was used as template. Water PCR Reagent (Sigma-Aldrich, Taufkirchen, Germany) served as a negative control and DNA from cell culture-derived *B. besnoiti* (Bb-EvoraCl2) tachyzoites was used as a positive control (Schares et al., 2008a). The reactions were performed in a thermal cycler (Eppendorf Mastercycler, Personal Thermal Cycler, Hannover, Germany) with an initial denaturation step of 95 °C for 5 min, followed by 35 cycles of denaturation (1 min at 95 °C), annealing (1 min at 54 °C) and extension (1 min at 72 °C), followed by a final extension step at 72 °C for 5 min. The amplification products were visualized after electrophoresis in 1.5% agarose gels stained with ethidium bromide. A 100 bp DNA ladder (Invitrogen GmbH, Karlsruhe, Germany) was used as a size standard. Amplicons were sequenced using a kit with 7-deaza-dGTP (ThermoSequenase™ DYEnamic Direct Cycle Sequencing Kit, GE Healthcare, München, Germany) and infrared dye (IRD)-700- and −800-labelled primers. The sequences of the IRD-labelled primers were the same as those used for PCR. Each sample was analysed in a DNA sequencer with a dual laser detection system (Long Readir LI-COR 4200 DNA Sequencer, MWG Biotech, Ebersberg, Germany). Sequences were assembled using the Lasergene 7.0 software (DNASTAR Inc., Madison, USA) and compared with sequences of *Besnoitia* spp. in GenBank™ by BLAST search.

### 2.8 Microsatellite amplification and sequencing

Microsatellite DNA sequences(Markers Bt-5, -6, -7, -9, -20, −21) were amplified by PCR using the primer pairs published by Madubata et al. (2012). PCR primers were used at a final concentration of 0.5 μM and dNTPs at 250 μM each (Amersham Biosciences, Piscataway, USA). InviTaq DNA polymerase (Bt-5, -6, -7, -9, -21; STRATEC molecular, Berlin, Germany) or DyNAzyme II DNA polymerase (Bt-20; Finnzymes, Espoo, Finland) was added at 5 or 2 U/25 μl, respectively, with the provided buffer. The reaction mix was supplemented with bovine serum albumin at a concentration of 20 μg/ml 1 μl of genomic DNA was used as template. Water PCR Reagent (Sigma-Aldrich, Taufkirchen, Germany) served as a negative control and DNA from cell culture-derived *B. besnoiti* (Bb1Evora03) tachyzoites was used as a positive control at a concentration of 10 ng/μl (Schares et al., 2008a). The reactions were performed in a thermal cycler (Eppendorf Mastercycler, Personal Thermal Cycler, Hannover, Germany) with an initial denaturation step of 95 °C for 5 min, followed by 10 cycles of denaturation (1 min at 95 °C), annealing (1 min at 52 °C with a decrement of 0.5 °C per cycle), 40 cycles of denaturation (1 min at 95 °C), annealing (1 min at 47 °C) and extension (1 min at 72 °C), and a final extension step at 72 °C for 10 min. In case of Bt-20, the initial denaturation step of 95 °C for 5 min was followed by 10 cycles of denaturation (1 min at 95 °C), annealing (1 min at 57 °C with a decrement of 0.5 °C per cycle), 40 cycles of denaturation (1 min at 95 °C), annealing (1 min at 52 °C) and extension (1 min at 72 °C), and a final extension step at 72 °C for 10 min. The amplification products were visualized in 1.5% agarose gels stained with ethidium bromide. A 100 bp DNA ladder (Invitrogen GmbH, Karlsruhe, Germany) was used as a size standard.

Amplification products were sequenced by two different protocols: (i) Amplicons were sequenced as described previously using a sequencing kit (Thermo Sequenase™ DYEnamic Direct Cycle, GE Healthcare, München, Germany) employing IRD-800-labelled primers with sequences published by Madubata et al. (2012), in a LI-COR DNA Sequencer 4200 (MWG Biotech, Ebersberg, Germany). (ii) PCR products were purified from agarose gels using a QIAquick gel extraction kit (QIAGEN) as recommended by the manufacturer and subjected to direct Sanger sequencing at GATC, Konstanz, Germany, on a 3730xl DNA analyzer (Applied Biosystems, Foster City, CA 94404, USA) using the microsatellite primers reported by Madubata et al. (2012). Microsatellite sequences were assembled using the Lasergene 7.0 software (DNA Star Inc., Madison, USA), compared with published sequences (Gutierrez-Exposito et al., 2016; Madubata et al., 2012) using the default parameters of ClustalW (Thompson et al., 1994).

### 2.9 Immunoblotting

Samples containing 2 × 10^6^ *Besnoitia* tachyzoites/lane were treated for 10 min at 94 °C with non-reducing sample buffer (2% (w/v) SDS, 10% (v/v) glycerol, 62 mM Tris HCl, pH 6.8). The parasite samples were electrophoresed in a 12.5% (w/v) SDS-polyacrylamide minigel together with marker proteins (LMW-SDS Marker Kit, GE Healthcare, Germany). Separated parasite antigens and marker proteins were electrophoretically transferred to polyvinylidene fluoride (PVDF) membranes (Immobilon-P, Millipore, Darmstadt, Germany) in a semi-dry transfer system, using a current of 1.5 mA/cm^2^ gel for 90 min. The part of the membrane coated with the marker proteins and a 0.5 mm wide strip of the antigen-coated part was cut off and the transferred proteins visualized using an India ink stain (Hancock and Tsang, 1983). The remaining antigen-coated membrane was blocked (30 min, room temperature) with PBS-TG (PBS, 0.05% [v/v] Tween 20, 2% [v/v] fish gelatine liquid [Serva, Heidelberg, Germany]), air-dried overnight, cut into 60 or fewer strips, which were stored frozen at −20 °C until used. Prior to incubation with diluted serum samples, the strips were blocked again with PBS-TG (30 min, room temperature). To detect antibodies against parasite antigens, the incubation of the strips with serum was performed as described by Schares et al. (1998) with few modifications. Sera diluted in PBS-TG were incubated with the strips for 60 min at room temperature. Sera (caribou sera, bovine sera) were diluted 1:100 with PBS-TG and incubated for 60 min (room temperature). Bovine positive control serum (positive) was the same as previously described (Schares et al., 2013). As a negative bovine control serum, a negative serum previously applied in *Neospora* tests was used (Schares et al., 2000). After washing in PBS-T (PBS, 0.05% [v/v] Tween 20), the strips were incubated with peroxidase conjugate solution (affinity purified goat anti-bovine IgG [H + L], Jackson ImmunoResearch Laboratories, West Grove, USA; diluted 1:500 in PBS-TG) for 60 min at room temperature. After washing in PBS-T and distilled water, antibody reactions were detected by adding substrate solution (40 μl H_2_O_2_ [30% (v/v)] and 30 mg 4-chloro-1-naphthol [Sigma] in 40 ml TBS, 20% [v/v] methanol). Relative molecular masses were determined by comparison with the LMW-SDS Marker standard. Mouse sera were analysed in the same way but using as a conjugate affinity purified rabbit anti-mouse IgG + IgM [H + L] (Jackson ImmunoResearch Laboratories, West Grove, USA; diluted 1:500 in PBS-TG).

## 3 Results

### 3.1 Cell cultivation and *in vitro* isolation of *B. tarandi*

*B. tarandi* MARC 145 cell cultures inoculated with mechanically released bradyzoites (1.0 × 10^4^; Fig. 1B) of the H-1145-skin (December 12^th^, 2011) showed multiplying parasites already two days after inoculation (December 14^th^, 2011). A first batch of parasite was cryo-preserved 10 days after the inoculation of the cell culture (December 22nd, 2011). The *B. tarandi* isolate was designated Bt-CA-Quebec1.

**Fig. 2:**
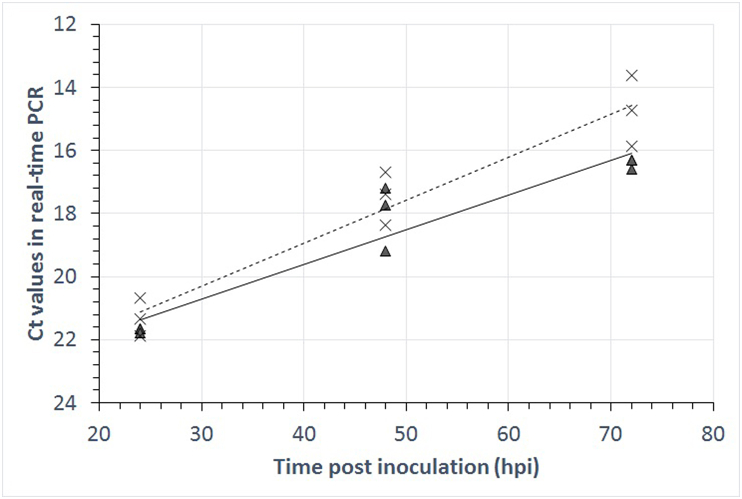
Growth of *Besnoitia besnoiti* Bb-EvoraCl2 (triangles) and *Besnoitia tarandi* Bt-CA-Quebec1 (crosses) as assessed by real-time PCR on *in-vitro* cultivated MARC-145 cells 24, 48 or 72 h p.i. Linear regression revealed that *B. tarandi* Bt-CA-Quebec1 grew faster than *B. besnoiti* BbEvoraCl2.

*In vitro* multiplication of the parasites in MARC-145 cells was assessed in 24 well plates inoculated in triplicate on day 0 with 1 × 10^6^ tachyzoites of *B. besnoiti* Bb-EvoraCl2 or *B. tarandi* Bt-CA-Quebec1. After 24, 48 or 72 h the experiment was terminated, the cells released mechanically, centrifuged and the pellets subjected to DNA extraction. Each sample was quantified for *B. besnoiti* DNA by BbRT2 real-time PCR (Schares et al., 2011). Bb-EvoraCl2 developed more slowly than Bt-CA-Quebec1 (Fig. 2). Assuming a PCR efficiency of about 95.7% (Schares et al., 2011), Bt-CA-Quebec1 had multiplied more than 120 times (i.e. 10^2.1^), while Bb-EvoraCl2 had done so about 50-times (i.e. 10^1.7^) within 72 h post inoculation (hpi).

### 3.2 Mouse experiments

Intraperitoneal inoculation of parasites mechanically released from H-1145 and H-1146 skin samples resulted in infection and signs of disease in 2/2 mice (H-1145) or 1/2 mice (H-1146), on days 8, 9 or 18 pi (Table 1). The peritoneal cavity of one mouse inoculated with 1.2 × 10^6^ parasites released from the H-1145 skin was flushed with 1 ml of cell culture medium; the retrieved fluid contained numerous tachyzoites (Mouse K280-2, 8 days pi). These tachyzoites (3.0 × 10^4^) were used to inoculate another two mice, which became sick on day 5 pi (Table 1). One of two mice that received mechanically isolated parasites from the skin of H-1146 (K281-2) and both two mice inoculated with pepsin-digested tissues (K282-1, K282-2), survived until day 42 pi and failed to seroconvert in *B. besnoiti* immunoblot. One H-1146 mouse (K281-1) showed signs of disease on day 18 pi. Parasites were seen in the peritoneal fluid of this animal and its tissues tested positive in the *Besnoitia* BbRT2 real-time PCR (Table 3).

**Table 3:** Real-time PCR results (Ct values in BbRT2 real-time PCR) for tissues of γ-interferon knockout mice after inoculation with *Besnoitia tarandi* bradyzoites obtained from Canadian caribou skin.

Tissue	Mouse ID (time after inoculation)					Median Ct over all positive animals
	K283-1 (5 dpi)	K283-2 (5 dpi)	K280-2 (8 dpi)	K280-1 (9 dpi)	K281 (18 dpi)	
Brain	22.1	27.1	27.1	22.9	22.9	22.9
Heart	20.1	24.9	24.9	22.9	19.1	22.9
Lung	13.6	19.3	19.3	15.8	17.0	17.0
Liver	20.8	24.0	24.0	N/A^a^	42.3	24.0
Kidney	45.5	39.6	39.6	34.9	25.4	39.6
Spleen	30.0	30.6	30.6	N/A	N/A	30.6
Skeletal muscle	20.2	23.1	23.1	20.6	22.3	22.3
Skin	29.1	27.2	27.2	34.1	21.8	27.2

a N/A = No amplification.

Overall, in PCR-positive mice, the organ with lowest Ct values (i.e. highest DNA concentration) was lung, followed by skeletal muscle, heart muscle and brain. The internal organs liver, kidney and spleen either tested negative or revealed high Ct values, except in the liver of animals necropsied on days 5–8 pi. Ct values in the skin were relatively high and ranged from 27.2 to 34.1, except for a mouse necropsied on day 18 pi (Table 3).

### 3.3 Genetic characterization

#### 3.3.1 Sequencing of ITS1 and rDNA gene fragments

A total of 2116 bases were sequenced, which cover the 3’-end of the 18S rRNA gene, the complete sequence of the ITS1 und the 5’-end of the 5.8S rRNA gene. The rDNA sequence was deposited in GenBank™ (accession number MH217579). Minor differences to rDNA gene fragments of *B. besnoiti* were observed; pairwise identities to sequences deposited for *B. besnoiti* ranged from 99.8% to 99.2%. These were located in both the 18S and the 5.8S rRNA gene. The ITS1 sequence was 100% identical with those deposited for *B. besnoiti* isolates in GenBank™, including isolates from Germany, Israel, Italy, Portugal, Spain and South Africa (FJ797432, DQ227420, JF314861, AY833646, EU789637, DQ227418, DQ227419, AF109678 [only 5.8S rDNA]). Relative to sequences deposited for related parasites like *B. bennetti* (AY665399 [18S rRNA gene and ITS1, complete sequence], *B. darlingi* (MF872603 [only 5.6S rDNA]) or *B. jellisoni* (AF291426 [only 5.6S rDNA]) pairwise identities of 99.3%, 99.2% or 99.0%, respectively, were observed.

#### 3.3.2 Microsatellite typing

Microsatellite typing by direct amplicon sequencing revealed in two out of six loci differences to patterns reported for *B. tarandi* from Northern Finland (Bt-6, Bt-7), from Finland/Oulu (Bt-7, Bt-21) and from Canada (Bt-6, Bt-7) (Table 4, Table 5). Regions flanking the repeat region were identical with previously reported ones (Madubata et al., 2012) with one exception in Bt-20 (Table 4): in this region, there was a deletion in the 5’–flanking regions and an insertion relative to the previously reported sequence (Madubata et al., 2012).

**Table 4:** *Besnoitia tarandi* Bt-CA-Quebec1 microsatellite sequences as determined by direct amplicon sequencing.

Microsatellite locus	Allele sequence, 5’ – 3’, repetitive sequences in brackets
Bt-5	GACGACGGCAGAG-(AC)_11_-GCAGACAAAGACAGAGCGCGCATGCGAATACAGA
Bt-6	GGACGGATACACACCTCGCAACAAATGAAGAGAACAAAACA-(AC)_12_-GAGAAAAAGCAGCTGCCGAATG
Bt-7	GGAGTCT-(TC)_6_-TGCAGTCGAAAAGAGACGCTC-(AC)_9_-GCGTCAACAATAACTTCCCTCAGCGAATGTGGA
Bt-9	GTCGAATCCTGTCCCCGTCT-(AC)_11_-TAGACCGCCTGCCGCGGACCCCGATCTCACGGCTGCCGAG
Bt-20^a^	TCCACGACAGTCCATCCGACAACACTATGCTCGCTAGTATACAAACTCATAC_AAAA**A**CG-(CA)_11_-AACACAAACACACGTTCTCCAGCGCGCCGTCCACCTGGGCAGCT
Bt-21	CGT-(CA)_23_-CGGAGCCCT

a Sequence differences in repeat flanking regions to sequences reported previously are marked bold.

**Table 5:** Microsatellite typing of isolates of *Besnoitia* species according to the number of repeat motifs in six microsatellite loci.

Besnoitia spp.	Isolate, sample	Host species	Country/Region	Micro-satellite locus						Reference
				Bt-5	Bt-6	Bt-7	Bt-9	Bt-20	Bt-21	
*B. tarandi*										
	Finland isolate	Reindeer	Finland/Oulu	11	12	8	11	11	24	(Dubey et al., 2004; Gutierrez-Exposito et al., 2016)
	Tissue samples	Reindeer	Finland/North	11	11	8	11	11	23	Madubata et al. (2012)
	Tissue samples	Caribou	Canada/Nunavut, Northwest Territories	11	11	8	11	11	23	Madubata et al. (2012)
	Bt-CA-Quebec1	Caribou	Canada/Quebec	11	12	9	11	11	23	This study
*B. bennetti*										
	Skin	Donkey	USA, Texas	12	13	8	8	8	6	(Gutierrez-Exposito et al., 2016; Ness et al., 2012)
		Donkey	USA, Michigan	12	13	8	8	8	6	(Elsheikha et al., 2005; Madubata et al., 2012)
*B. besnoiti*										
	Bb-Israel	Cattle	Israel/Golan Heights	10/9	12	8/9	10	8	13/12	(Frey et al., 2016; Gutierrez-Exposito et al., 2016; Madubata et al., 2012)
	Bb-Ger1	Cattle	Germany/Bavaria	10	12	8	10	8	13	(Gutierrez-Exposito et al., 2016; Schares et al., 2009)
	Bb-Italy2	Cattle	Italy/Emilia Romagna Apennines	10	12	8	10	8	15	(Frey et al., 2016; Gutierrez-Exposito et al., 2016)
	Bb-Spain1, Bb-Spain2	Cattle	Spain/Guadalajara, Huesca	10	12	8	10	8	13	(Frey et al., 2016; Gutierrez-Exposito et al., 2016)
	Bb-France	Cattle	French Pyrenees	10	12	8	10	8	13	(Frey et al., 2016; Gutierrez-Exposito et al., 2016)

### 3.4 Antigenic characterization by immunofluorescence and immunoblotting

A serum collected from a rabbit that had been subcutaneously inoculated with *B. besnoiti* tachyzoites, and bled 11 weeks post infection (Basso et al., 2011) showed strong reactions with *B. tarandi* tachyzoites multiplying in MARC-145 cells (Fig. 1C).

**Fig. 3:**
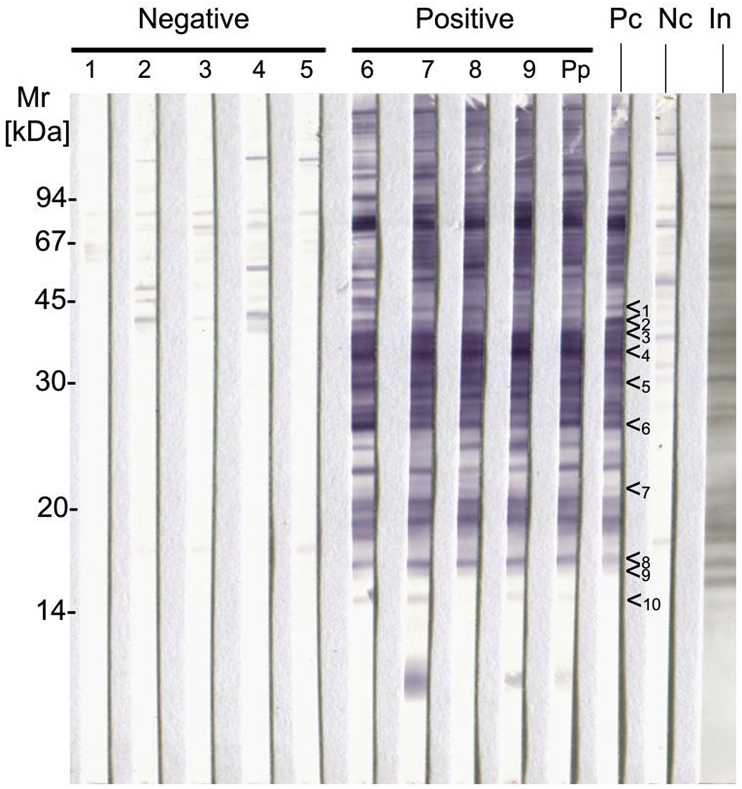
Reactivity of *B. tarandi*-positive caribou sera with *B. besnoiti* Bb1Evora03 antigen. Patterns resemble the picture obtained with bovine besnoitiosis sera. Sera from caribou without (Negative, sera 1–5) or with clinical signs of besnoitiosis (Positive, sera 6–9, including the pool of these sera Pp) were tested. A serum from a *Besnoitia besnoiti*-infected cattle was used as a positive control (Pc) and a *B. besnoiti*-negative serum as a negative control (Nc). On the right side, an India ink staining (In) of the antigen is displayed. Close to the strip that had been probed with the *B. besnoiti* positive bovine control serum, the locations of 10 bands are marked (<_1-10_), which have previously been used to identify specific reactions against *B. besnoiti* (Schares et al., 2010).

Four sera collected from caribou with clinical signs of besnoitiosis (tissue cysts in scleral conjunctivae and in the skin) and five sera from caribou without clinical signs were examined by immunoblot using *B. besnoiti* Bb1Evora03 antigen in comparison with a bovine *B. besnoiti-*positive bovine serum. To evaluate the immunoblot, the scoring system published for *B. besnoiti* was applied (Schares et al., 2010) focussing on the reactivity against a set of 10 antigen bands (Fig. 3, bands 1–10). All positive caribou (including a pool consisting of equal volumes from the four positive sera) showed reactions with all 10 *B. besnoiti* bands previously selected to identify a *B. besnoiti*-positive reaction (Fig. 3). Analysis of negative caribou sera revealed only minor reactions with bands that did not co-migrate with the *B. besnoiti* specific bands (Fig. 3).

**Fig. 4:**
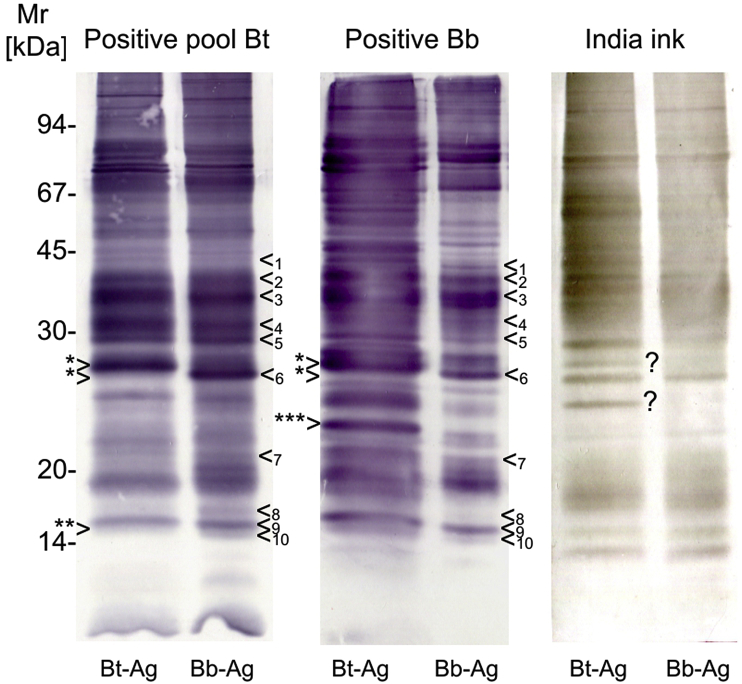
*B. tarandi* Bt-CA-Quebec1 (Bt-Ag) and *B. besnoiti* Bb1Evora03 (Bb-Ag) antigens differ slightly. A pool of *B. tarandi*-positive caribou sera (Positive pool Bt) and a serum from a bovine besnoitiosis case (Positive Bb) were tested with *B. tarandi* Bt-CA-Quebec1 and *B. besnoiti* Bb1Evora03 antigens. The following differences were observed: (i) an apparent shift in antigen band 6 (<_6_), which has a higher relative molecular weight in *B. tarandi* Bt-CA-Quebec1 than in *B. besnoiti* Bb1Evora03; (ii) in the India ink protein staining (India Ink), additional bands seem to be present in the *B. tarandi* Bt-CA-Quebec1 antigen (?); (iii) differences in the reactivity of sera were marginal, e.g. the positive Bt serum failed to react (**>) with antigen band 10 (<_10_), while the positive Bb serum recognized this band; (iv) the positive Bb serum reacted much stronger with a ∼23 kDa protein in the *B tarandi* Bt-CA-Quebec1 antigen (***>), while a similar reactivity was missing in the *B. besnoiti* Bb1Evora03 antigen.

In a second set of experiments, the positive caribou pool and a *B. besnoiti* bovine positive control serum were probed with *B. tarandi* Bt-CA-Quebec1 and *B. besnoiti* Bb1Evora03 antigen. With the *B. besnoiti* Bb1Evora03 antigen, the positive caribou pool serum showed a pattern that was similar to the *B. besnoiti* bovine positive control serum, i.e. all 10 *B. besnoiti* specific bands were recognized. In the caribou antigen, only one major difference was observed; the specific band no. 6 was missing and was replaced by a strong reacting band with a slightly higher relative molecular weight, suggesting a shift (marked by *> in Fig. 4). After India ink protein staining, additional bands appeared in the *B. tarandi* Bt-CA-Quebec1 antigen in comparison with *B. besnoiti* Bb1Evora03 (marked by ? in Fig. 4). One of these bands co-migrated with a band recognized by the pooled positive caribou serum and the *B. besnoiti* positive bovine serum. Minor differences in the reactivity of the positive *B. tarandi* Bt-CA-Quebec1 serum and the bovine besnoitiosis serum were observed: the positive caribou pool failed to react (Fig. 4; **>) with antigen band 10 (Fig. 4; <10). Moreover, the positive bovine *B. besnoiti* serum reacted much stronger with a ∼23 kDa Mr protein in the *B tarandi* Bt-CA-Quebec1 antigen (Fig. 4; ***>) while a similar reactivity was missing in the *B. besnoiti* Bb1Evora03 antigen.

## 4 Discussion

Besnoitiosis is an emerging disease in caribou herds in Quebec and Labrador, Canada (Kutz et al., 2009). In the present study, we report on the *in vitro*-isolation of *B. tarandi.* To our knowledge, we obtained the first *in vitro* isolate of *B. tarandi* from North America and from caribou (*Rangifer tarandus caribou*), and the second *in vitro*-isolate of *B. tarandi* altogether so far. The *in vitro* isolate was obtained directly from the skin of a Canadian woodland caribou of the migratory ecotype. This contrasts with our previous experience with the isolation of *B. besnoiti* where previous passages in γ-interferon knockout mice were necessary (Schares et al., 2009). However, for *B. tarandi* (Dubey et al., 2004) and also for *B. besnoiti*, other groups also succeeded in the direct isolation without prior mouse passages into cell culture (Basso et al., 2013; Cortes et al., 2006; Fernandez-Garcia et al., 2009; Shkap et al., 1987). Thus, the direct *in vitro* isolation does not seem to be a *B. tarandi*-specific trait.

The new *in vitro*-isolate was named Bt-CA-Quebec-1. Sequencing of the 3’-end of the 18S rRNA gene, the complete ITS1 gene and the 5’-end of the 5.8S rRNA gene revealed only minor differences to rDNA gene fragments of *B. besnoiti*. The ITS1 sequence was 100% identical or almost 100% identical to those deposited for *B. besnoiti* and *B. bennetti* isolates in GenBank™ including isolates from Germany, Israel, Italy, Portugal, Spain, South Africa and USA. This shows the close phylogenetic relationship between *B. besnoiti, B. tarandi and B. bennetti*, which had already been observed in a number of studies (Dubey et al., 2004; Madubata et al., 2012; Schares et al., 2011); this very close relationship was also noted, when the ribosomal DNAs of *B. besnoiti* and *B. caprae* were compared (Namazi et al., 2011). Consequently, rDNA based PCRs established for *B. besnoiti* (Cortes et al., 2007; Schares et al., 2011) may also be suitable for a PCR diagnosis of *B. tarandi*. This holds also true for the real-time PCR BbRT2 applied here (Schares et al., 2011).

*Besnoitia besnoiti* infections have been proved in wild ruminant species such as red deer (*Cervus elaphus*) and roe deer (*Capreolus capreolus*) in those regions where bovine besnoitiosis is endemic (Arnal et al., 2017; Gutierrez-Exposito et al., 2016). The close phylogenetic relationship of *B. tarandi* with *B. besnoiti* may trigger a discussion on the host specificity of *B. tarandi* and on the possibility that *B. tarandi* could be transmitted to cattle. Cohabitation experiments with *Besnoitia*-infected caribou and uninfected cattle and mule deer were performed in the past; there was no clinical evidence from this experiment indicating that *B. tarandi* may be transmitted to cattle or mule deer (Lewis, 1992). However, the assessment was based on the observation of clinical signs and light microscopic examination. A subclinical infection with a low number of tissue cysts in cattle and mule deer might have remained unnoticed. As other species of Canadian wildlife like muskoxen (Gunn et al., 1991) and mule deer (Glover et al., 1990) have been reported to suffer from besnoitiosis and *Besnoitia*-positive antibody responses were detected in Canadian muskoxen and in a bison (*Bos bison*; Gutierrez-Exposito et al. (2012)), the host-specificity of this parasite needs to be further investigated. To assess the host-specificity, novel serological and DNA detection tools are needed, similar to those used in cohabitation experiments in cattle (Gollnick et al., 2015). In addition, experimental infections of cattle with *B. tarandi* may help to assess intermediate host specificity. In any case, it has been questioned whether more *Besnoitia* species are present in roe deer, mule deer and muskoxen, which belong to different genera (Dubey et al., 2004). Further, it was regarded as uncertain, if the same parasite species affects both reindeer and caribou (Dubey et al., 2004). Therefore, the *in vitro* isolation of *B. tarandi* from caribou is important to compare isolates from reindeer and caribou at the genetic level.

At the moment, the only sufficiently validated and robust way to differentiate *B. tarandi* from *B. besnoiti* genetically is to identify differences by analyzing microsatellite loci (Madubata et al., 2012). Especially, the loci Bt-20 and Bt-21 appear to be helpful (Table 5). In addition, a separation of *B. tarandi* from *B. bennetti* (a *Besnoitia* sp. circulating for example in North American donkeys (Ness et al., 2012)) seems to be possible. Again, the loci Bt-20 and Bt-21 appear to be suitable (Madubata et al., 2012). Surprisingly there were also different typing results in the loci Bt-6 and Bt-7 for Bt-CA-Quebec1 and those obtained for skin samples from caribou sampled in the Canadian territories Nunavut and Northwest Territories (Table 5). This could be regarded as an indication that differences might exist between *B. tarandi* in caribou from different regions. It also suggests that the recent outburst of this parasite, unreported in the eastern populations prior to 2006, did not stem from a western population source. Based on recent findings on the existence of different caribou lineages (Yannic et al., 2017), one could hypothesize that different *B. tarandi* linages co-evolved together with caribou. To test this, larger studies in different caribou populations are necessary and several samples or isolates per caribou lineage need to be obtained.

Data on a single *B. tarandi* isolate and two Iberian *B. besnoiti* isolates revealed a limited number of nucleotide polymorphisms in a protein disulphide isomerase gene (Arnal et al., 2017). Further studies a necessary to confirm these nucleotide polymorphisms, e.g. by making use of recently published whole-genome sequencing (WGS) data on *B. besnoiti* (Schares et al., 2017b) and future *B. tarandi* WGS data single nucleotide polymorphisms (SNP) analyses. If confirmed, polymorphisms similar to those described by Arnal et al. (2017) may prove as a suitable tool to differentiate the two species.

The γ-interferon knockout mice used in this study were highly susceptible to infection with *B. tarandi* bradyzoites and tachyzoites of Bt-CA-Quebec1, which is similar to the situation regarding *B. besnoiti* (Schares et al., 2009). *B. tarandi* bradyzoites obviously underwent a rapid stage conversion and mice inoculated intraperitoneally with 1.2 × 10^6^ bradyzoites fell ill 8–9 days pi. Mice sub-inoculated intraperitoneally with mouse-derived tachyzoites (although at a considerably lower dose of 3.0 × 10^4^) fell ill 5 days pi. Previous experiences with another *B. tarandi* isolate were similar and γ-interferon knockout mice developed disease after 9–26 days pi, if undigested tissues or tachyzoites were applied subcutaneous or orally (Dubey et al., 2004). In experiments, where trypsin-digested tissues were used, the time between inoculation and disease was similar or about one week longer (Dubey et al., 2004). In the present study, we tried to use the standard pepsin-digestion protocol to release bradyzoites from tissues. However, this method, which is often successfully applied to release *Toxoplasma gondii* bradyzoites from tissue cysts (Dubey, 1998; Schares et al., 2017a), failed in the case of the *B. tarandi* bradyzoites and only the mechanically released bradyzoites caused disease in γ-interferon knockout mice. The pepsin-digestion method possibly needs refinement before it is successfully applied to *B. tarandi* tissue cysts. Moreover, it has to be established, whether bradyzoites of *B. tarandi* are more resistant to pepsin-digestion than *B. tarandi* tachyzoites. If this is not the case, this has an implication for the role of tissue cysts in the life cycle of *B. tarandi.* If they are not resistant to pepsin-digestion, bradyzoites are unlikely to survive the gastric passage in a carnivorous definitive host.

The organ with the highest *B. tarandi* DNA loads was the lung of γ-interferon knockout mice. This corroborates previous finding in γ-interferon knockout mice inoculated with *B. besnoiti* (Gentile et al., 2012). In previous experiments with *B. tarandi*, tachyzoites were seen in many organs, but they were most abundant in the liver, the lungs and in the spleen (Dubey et al., 2004). Lung was the predilection organ in our study, but liver and spleen were not identified as such, as real-time PCR results were very variable and even in some animals negative in these organs (Table 3). Our observation that only one skin sample of a mouse necropsied late (i.e. on day 18 pi), contained parasitic stages suggests that skin may become more important as a predilection site for *B. tarandi* parasitic stages after prolonged duration of infection.

In our hands, *B. tarandi* Bt-CA-Quebec1 rapidly grew in MARC-145 cells. To our surprise, in our preliminary experiments *B. tarandi* Bt-CA-Quebec1 grew faster than our laboratory standard *B. besnoiti* clone (Bb-EvoraCl2). A similar experience was previously made in trials on invasion and intracellular proliferation of various *B. besnoiti* isolates including also *B. tarandi* isolate from reindeer (*R. t. tarandus*) obtained in Finland (Frey et al., 2016). In their study, *B. besnoiti* (Bb-Israel) and *B. tarandi* isolates were the most prolific, as determined by the tachyzoite yields 72 h pi (Frey et al., 2016).

We used *in vitro* growing *B. tarandi* Bt-CA-Quebec1 and *B. besnoiti* Bb1Evora03 tachyzoites as antigen in the immunoblot, to compare the differences in reactivity using Besnoitia positive caribou sera. No major differences were observed, suggesting that *B. tarandi* and *B. besnoiti* tachyzoites have more or less the same antigenic composition. This corroborates observations reported by others (Garcia-Lunar et al., 2014; Gutierrez-Exposito et al., 2012). The situation seems to be very similar in *B. bennetti*, as *B. bennetti* infected donkeys and *B. besnoiti* infected cattle showed analogous reaction patterns with *B. besnoiti* antigens (Ness et al., 2014).

However, *B. tarandi* Bt-CA-Quebec1 strain seems to express some of antigens differentially to *B. besnoiti* Bb1Evora03. In a few bands, clear differences between Bt-CA-Quebec1 and Bb1Evora03 were observed, suggesting that some potentially diagnostic proteins are expressed in a different way in Bt-CA-Quebec1. In the case of the most prominent difference (diagnostic antigen 6), there seemed to be a clear shift in the apparent relative molecular weight, which needs further confirmation, e.g. by using antigen specific monoclonal antibodies similar to those recently established by Garcia-Lunar et al. (2018). Whether the differences in antigen composition are unique to particular isolates of *B. tarandi* or *B. besnoiti*, or represent more general differences, i.e. differences between species, has to be clarified.

In conclusion, we report for the first time an *in vitro* isolation of *B. tarandi* from caribou (*R. t. caribou*). This complements a previous isolation of *B. tarandi* from reindeer (*R. t. tarandus*) (Dubey et al., 2004) and allows future comparative molecular and phylogenetic studies. Due to its fast proliferation *in vitro*, the Bt-CA-Quebec1 isolate may represent an antigen source to establish *B. tarandi*-specific serological tools and to study the biology and the epidemiology of this parasite species in North American caribou.

## Conflicts of interest

The authors report no conflict of interest.
