# Supplementary material for: Besnoitia tarandi in Canadian woodland caribou – Isolation, characterization and suitability for serological tests
Source: Int J Parasitol Parasites Wildl. 2018 Nov 27;8:1–9. doi: 10.1016/j.ijppaw.2018.11.005 (PMC6280010; doi:10.1016/j.ijppaw.2018.11.005)
Supplement: Supplementary data [file mmc1.doc]

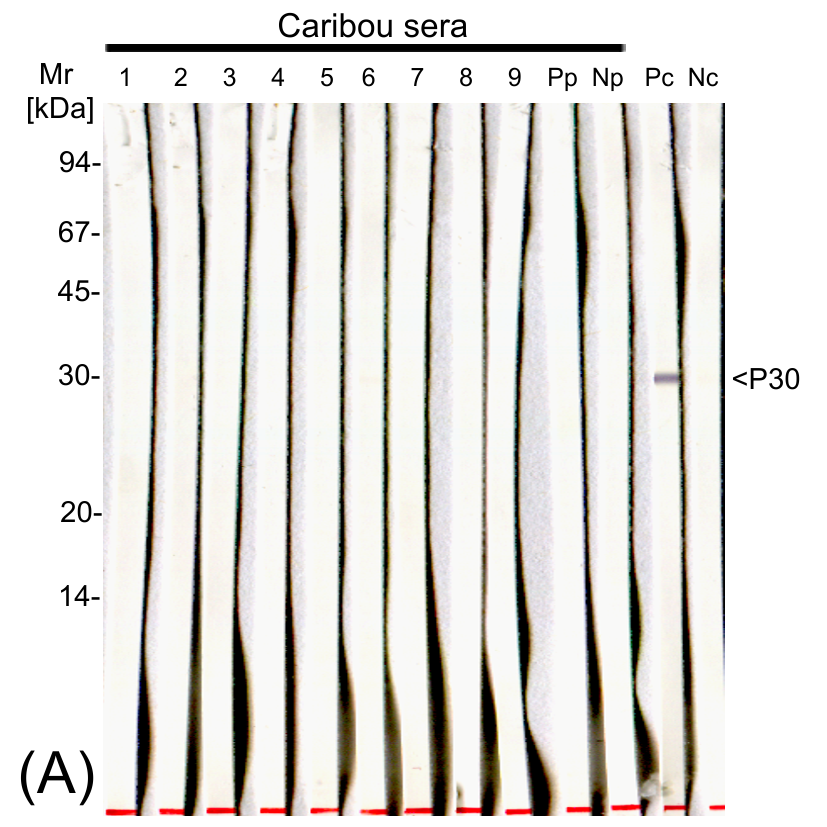

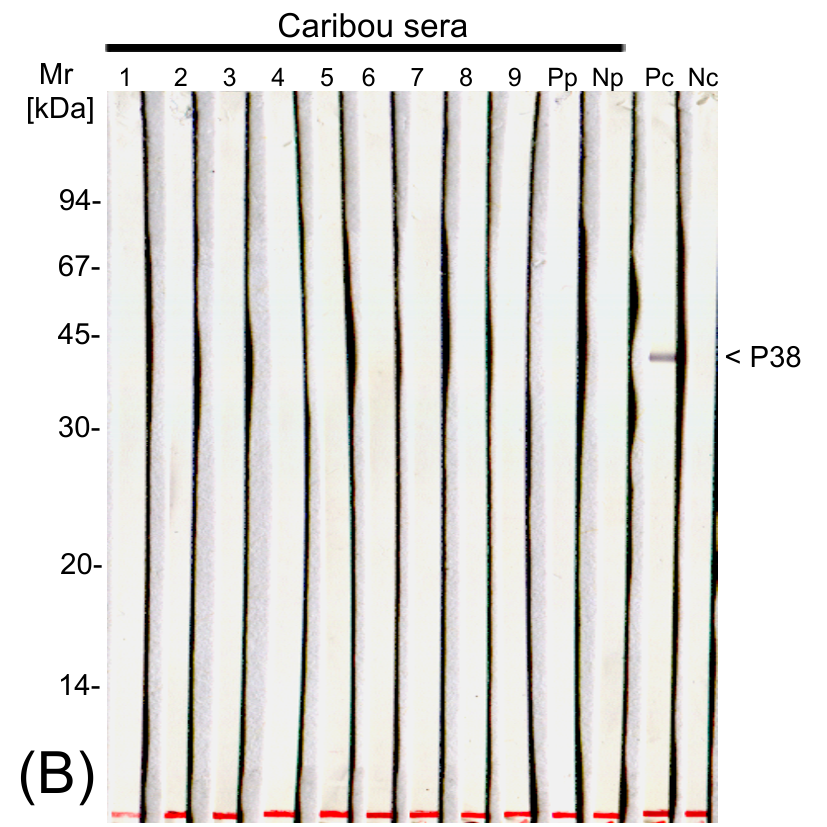


**Figure S1:** Serological examination of caribou sera for the presence of antibodies against immunodominant antigens of (A) *Toxoplasma gondii* (p30; TgSAG29B) and (B) *Neospora caninum* (p38, NCSRS2). None of the caribou sera showed specific reactions. 1-5: *Besnoitia* negative caribou sera: BCN-01, 03, 07, 09, 11; 6-9: *Besnoitia* positive caribou sera: F217, F216, F211, F208; Pp: *Besnoitia* positive caribou pool; Np: *Besnoitia* negative caribou pool; Pc: Positive ovine serum (*T. gondii*) or bovine serum (*N. caninum*); Nc: Negative ovine serum (*T. gondii*) or bovine serum (*N. caninum*) serum.

**Method:**

The RH strain of *T. gondii* (Sabin, 1941) and the NC-1 strains of *N. caninum* (Dubey et al., 1988) were maintained in MARC-145 cell cultures and purified as previously described (Schares et al., 1999; Schares et al., 1998). Cell culture derived tachyzoites were frozen as a pellet at –80 °C until used for immunoblot or antigen purification. The surface antigens p30 (TgSAG29B) of *T. gondii* and p38 of *N. caninum* were purified by affinity-chromatography essentially as described, using the monoclonal antibodies P30/3 (Hosseininejad et al., 2009) and 4.15.15 (Schares et al., 2000). SDS-PAGE and immunoblots were performed as described (Schares et al., 2000). Briefly, purified p30 or p38 (0.5 µg) were incubated in non-reducing sample buffer (2 %[w/v] sodium dodecyl sulfate (SDS), 10 %[v/v] glycerol, 62 mM TrisHCl, pH 6.8) for 1 min (94°C), separated in 12%[w/v] SDS polyacrylamide minigels of 60 x 70 x 1 mm size and transferred to PVDF membranes (Immobilon-P, Millipore). After the transfer, membranes were blocked using PBS-TG (PBS with 0.05 % (v/v) Tween 20 (Sigma) and 2% (v/v) liquid fish gelatine (Serva, Germany)), cut into 50 stripes and examined as described below. Sera were diluted 1:100 in PBS-TG. The reactivity of sera with a single band of 30 kDa Mr (*T. gondii*) or 38 kDa Mr (*N. caninum*) was visualized using a peroxidase conjugated anti-bovine IgG (H + L) (Jackson Immunoresearch Laboratories, West Grove, PA, USA) diluted 1:500 in PBS-TG.

**References:**

Dubey, J.P., Hattel, A.L., Lindsay, D.S., Topper, M.J., 1988. Neonatal *Neospora caninum* infection in dogs: isolation of the causative agent and experimental transmission. J. Am. Vet. Med. Assoc. 193, 1259-1263.

Hosseininejad, M., Azizi, H.R., Hosseini, F., Schares, G., 2009. Development of an indirect ELISA test using a purified tachyzoite surface antigen SAG1 for sero-diagnosis of canine *Toxoplasma gondii* infection. Vet. Parasitol. 164, 315-319.

Sabin, A., 1941. Toxoplasmic encephalitis in children. Journal of the American Medical Association 116, 801-814.

Schares, G., Dubremetz, J.F., Dubey, J.P., Bärwald, A.L., A., Conraths, F.J., 1999. *Neospora caninum*: Identification of 19-, 38-, and 40-kDa surface antigens and a 33-kDa dense granule antigen using monoclonal antibodies. Exp. Parasitol. 92, 109-119.

Schares, G., Peters, M., Wurm, R., Bärwald, A.C., F. J., 1998. The efficiency of vertical transmission of *Neospora caninum* in dairy cattle analysed by serological techniques. Vet. Parasitol. 80, 87-98.

Schares, G., Rauser, M., Söndgen, P., Rehberg, P., Bärwald, A., Dubey, J.P., Edelhofer, R., Conraths, F.J., 2000. Use of purified tachyzoite surface antigen p38 in an ELISA to diagnose bovine neosporosis. Int. J. Parasitol. 30, 1123-1130.
